# Cinical, Metabolic, and Genetic Analysis and Follow-Up of Eight Patients With *HIBCH* Mutations Presenting With Leigh/Leigh-Like Syndrome

**DOI:** 10.3389/fphar.2021.605803

**Published:** 2021-03-08

**Authors:** Junling Wang, Zhimei Liu, Manting Xu, Xiaodi Han, Changhong Ren, Xinying Yang, Chunhua Zhang, Fang Fang

**Affiliations:** ^1^ Department of Neurology, Beijing Children's Hospital, Capital Medical University, National Center for Children's Health, Beijing, China; ^2^ Department of Research, Development of MILS International, Ishikawa, Japan

**Keywords:** HIBCH gene, Leigh/Leigh-like syndrome, C4-OH, 2,3-dihydroxy-2-methylbutyrate (23DH2MB), mitochondrial disorders, children

## Abstract

3-Hydroxyisobutyryl-CoA hydrolase (*HIBCH*, NM_014362.3) gene mutation can cause HIBCH deficiency, leading to Leigh/Leigh-like disease. To date, few case series have investigated the relationship between metabolites and clinical phenotypes or the effects of treatment, although 34 patients with *HIBCH* mutations from 27 families have been reported. The purpose of this study was to analyze the phenotypic spectrum, follow-up results, metabolites, and genotypes of patients with HIBCH deficiency presenting with Leigh/Leigh-like syndrome and explore specific metabolites related to disease diagnosis and prognosis through retrospective and longitudinal studies. Applying next-generation sequencing, we identified eight patients with *HIBCH* mutations from our cohort of 181 cases of genetically diagnosed Leigh/Leigh-like syndrome. Six novel *HIBCH* mutations were identified: c.977T>G [p.Leu326Arg], c.1036G>T [p.Val346Phe], c.750+1G>A, c.810-2A>C, c.469C>T [p.Arg157*], and c.236delC [p.Pro79Leufs*5]. The Newcastle Pediatric Mitochondrial Disease Scale (NPMDS) was employed to assess disease progression and clinical outcomes. The non-invasive approach of metabolite analysis showed that levels of some were associated with clinical phenotype severity. Five (5/7) patients presented with elevated C4-OH in dried blood spots, and the level was probably correlated with the NPMDS scores during the peak disease phase. 2,3-Dihydroxy-2-methylbutyrate in urine was elevated in six (6/7) patients and elevated S-(2-caboxypropyl)cysteamine in urine was found in three patients (3/3). The median age at initial presentation was 13 months (8–18 months), and the median follow-up was 2.3 years (range 1.3–7.2 years). We summarized and compared with all reported patients with *HIBCH* mutations. The most prominent clinical manifestations were developmental regression/delay, hypotonia, encephalopathy, and feeding difficulties. We administered drug and dietary treatment. During follow-up, five patients responded positively to treatment with a significant decrease in NPMDS scores. Our research is the largest case series of patients with *HIBCH* mutations.

## Introduction

Leigh syndrome (LS), as the most common presentation of mitochondrial disorders in children, is a devastating neurodegenerative disease accompanied by symmetrical lesions in basal ganglia, brainstem, and with genetic and clinical heterogeneity. It sometimes presents in patients with normal laboratory findings, atypical neuroimaging or symptoms, but highly suggestive of LS, which is known as Leigh-like syndrome (Baertling et al., 2014). It is related to over 95 disease-associated genes and still in expanding scenery (Lake et al., 2016; Rahman and Rahman, 2018; Baldo and Vilarinho, 2020). LS has an estimated incidence of 1:40,000 newborns (Rahman et al., 1996). There is no research on the incidence of LS in Chinese. Currently, there is no standard treatment for LS and it is very challenging to performing large-scale clinical research, due to the variable phenotypes, genetic heterogeneity and early death.

**FIGURE 1:**
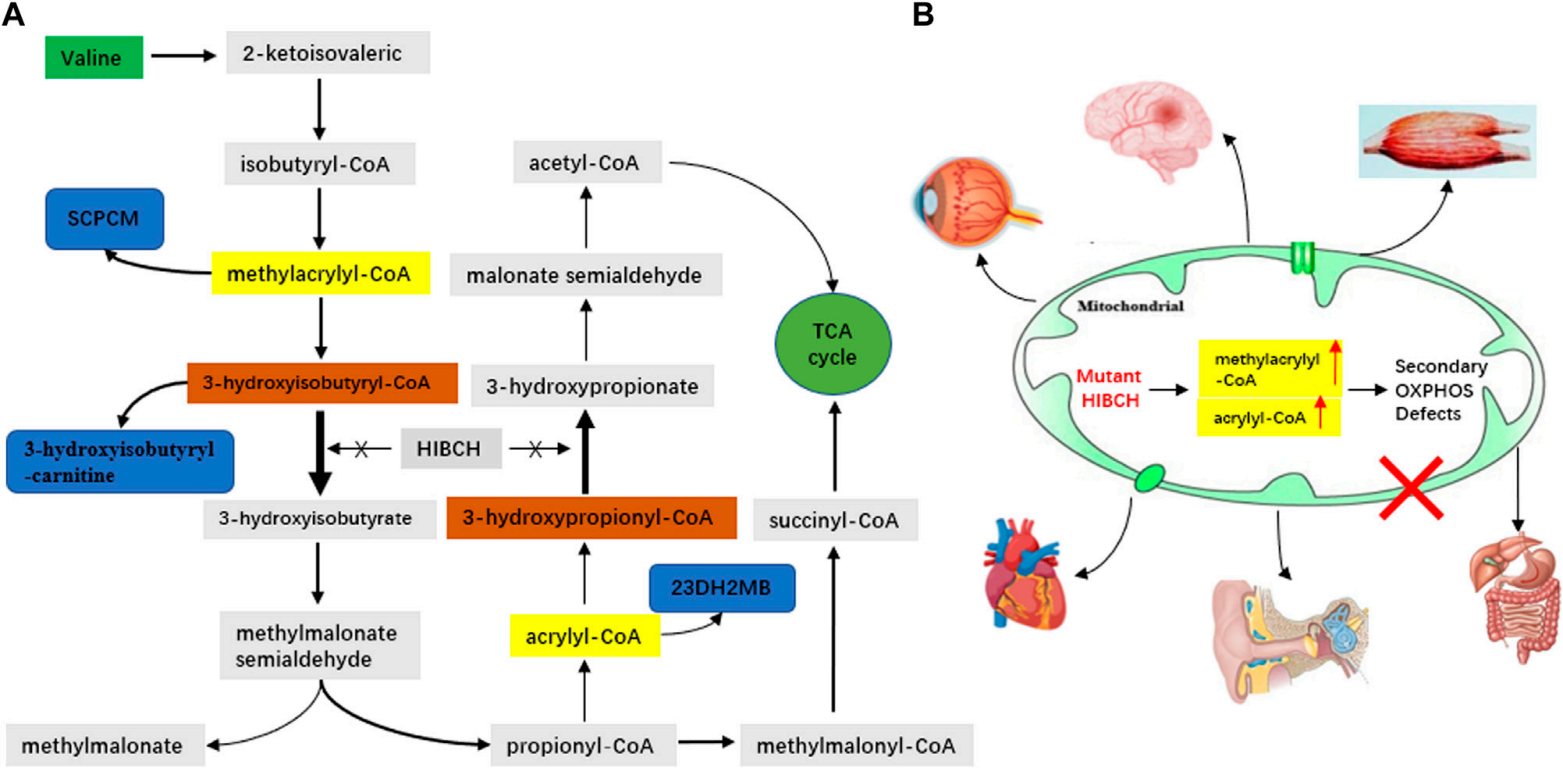
Schematic representation of the valine metabolic pathway involved in HIBCH **(A)**, and mechanism of HIBCH deficiency with the resulting clinical phenotypes **(B)**. SCPCM, S-(2-caboxypropyl)cysteamine; 23DH2MB, 2,3-dihydroxy-2-methylbutyrate.

The mitochondrial nuclear gene *HIBCH* (cytogenetic location: 2q32.2; GenBank accession number: NM_004092.3; OMIM* 610690), encodes the 3-hydroxyisobutyryl-CoA hydrolase (HIBCH) protein. It is mainly responsible for converting 3-hydroxyisobutryl-CoA to 3-hydroxyisobutyric acid in valine catabolism, as well as 3-hydroxypropanoyl-CoA to 3-hydroxypropionic acid, a hypothetical minor pathway for propionic acid catabolism, which feeds into the valine catabolic pathway (Figure 1A) (Shimomura et al., 1994; Peters et al., 2014). *HIBCH* mutations were first identified to cause HIBCH deficiency (HIBCHD, OMIM #250620) in 2007 (Loupatty et al., 2007), and it was reported that it could lead to Leigh/Leigh-like disease due to secondary oxidative phosphorylation (OXPHOS) defects (Figure 1B) (Ferdinandusse et al., 2013; Yamada et al., 2014; Soler-Alfonso et al., 2015; Stiles et al., 2015).

The most comprehensive case series of *HIBCH* mutations was a report of five Turkish cases from two unrelated families (Schottmann et al., 2016). To date, just 31 *HIBCH* gene mutations have been reported, with 34 patients on a broad phenotypic spectrum ranging from early death (Brown et al., 1982; D’Gama et al., 2020) to just movement disorder with survival into adulthood, although the estimated incidence of HIBCH deficiency was 1 in ∼130,000 individuals (Stiles et al., 2015). The current understanding and detection of *HIBCH* mutations are insufficient. There is a lack of systematic research on the clinical, genotype, and biochemical characteristics of patients with *HIBCH* mutations. In the early stage, detection of HIBCH enzymatic activity and respiratory chain enzymes in patients’muscle or skin tissues can help clinicians make a diagnosis. However, enzymatic tests of biopsied tissue is an invasive method and sometimes yields inconsistent results (Loupatty et al., 2007; Reuter et al., 2014), limiting their clinical application. At present, genetic testing for disease diagnosis is more widespread. We propose that non-invasive metabolites should be investigated to assist early diagnosis and timely treatment.

Herein, we analyzed the genetic, metabolic, and clinical features and follow-up findings of 8 Chinese patients with *HIBCH* gene mutations identified by next-generation sequencing (NGS) combined with metabolic examination and who presented with Leigh/Leigh-like Syndrome.

## Methods

### Patients

Eight patients with identified *HIBCH* mutations who were from our large cohort of case series with 181 cases of genetically diagnosed Leigh/Leigh-like syndrome were recruited to participate in this study from October 2012 to December 2019.

### Genetic Analyses

Due to different times of recruitment, we applied different NGS approaches: targeted panel sequencing (Patients 1 and 2) (Fang et al., 2017) and whole-exome sequencing (WES) (Patients 3, 4, 5, 6, 7, and 8). We performed Sanger sequencing to validate the identified *HIBCH* mutations and test the parental origin of available family members.

Briefly, genomic DNA was extracted from peripheral blood using TIANamp Blood DNA Kit (Tiangen, Beijing, China). Then 200 ng high-quality genomic DNA were utilized for library preparation. Genomic DNA was enzymatic sheared, end repaired, phosphorylation of the 5′ prime ends, a-tailing of the 3′ends and ligated to sequencing adapters, then PCR amplified following standard library preparation SOP, by using KAPA Hyper Plus Kits (Kapa Biosystems, Wilmington, MA, United States). The post-PCR libraries were captured by SureSelectXT Human All Exon V6 (Agilent, United States) or clinical exome analysis (6110 genes) (Agilent, United States), respectively. The final enriched libraries were sequenced with 2 × 150 bp on Illumina sequencers (Illumina, San Diego, CA, United States). The average sequencing depth for each sample was 149.3X (Patient 1), 174.9X (Patient 2), 148.7X (Patients 3 and 4), 133.9X (Patient 5), 130.5X (Patient 6), 132.02X (Patient 7), and 130.2X (Patient 8). Sequence data were aligned to the human genome reference (UCSC Genome Browser build hg19) using Burrows-Wheeler Aligner (BWA) (Li, 2012). Variant filtering and annotation was carried out with ANNOVAR software (Wang et al., 2010). Variants were screened as follows: 1) Preference to the variants related to the diseases, small INDEL, canonical splice sites, and nonsense variants. 2) Minor allele frequency (MAF) in normal populations <5% (except for known MAF ≥5% pathogenicity). 3) Preference to variants in the Human Gene Mutation Database (HGMD), ClinVar. 4) Preference to variants in the Online Mendelian Inheritance in Man database. Pathogenic variants were defined according to Standards and guidelines for the interpretation of sequence variants published by the American College of Medical Genetics (ACMG) in 2015 with Human Genome Variation Society nomenclature (Richards et al., 2015).

Additionally, The deletion at 2q32.2 including *HIBCH* gene was firstly identified by WES as the loss of heterogeneity in the Patient 8. Droplet-based digital PCR (ddPCR) was performed in the patient and his father to verify this deletion. Using QX200™ Droplet digital™ PCR systems, we performed the processes of sample dispersion, amplification and Quantification in turn. The number of droplets was obtained in the droplet reader based on the fluorescence amplitude (Hindson et al., 2011). And the data was analyzed by the software QuantaSoft™ analysis Pro 1.0.596.

### Metabolite Measurements

Amino acids and acylcarnitines in dried blood spots (DBS) were analyzed by electrospray tandem mass spectrometry (LC-MS/MS) for C4-OH (composed of 3-hydroxyisobutyryl-carnitine, 3-hydroxy-butyryl-carnitine, and malonylcarnitine [C3-DC]). Samples were prepared as follows: a 3-mm diameter disc was punched in a microtiter plate, 100 μl internal standard solution was added, and the plate was covered. After gentle shaking at 45°C for 45 min, the solvent after elution was transferred into a new microtiter plate and analyzed immediately. An LCMS-8040 (Shimadzu, Japan) in positive mode was utilized for acylcarnitines and amino acids analysis. For MS/MS data acquisition, the peak including C4-OH (248.2>85) was observed with multiple reaction monitoring.

Seven patients’ urine metabolites, including 2,3-dihydroxy-2-methylbutyrate (23DH2MB), were analyzed by standard gas chromatography-mass spectrometry (GC-MS). Briefly, 100 μl urine was incubated with 40 units urease at 37°C for 15 min. After adding an internal standard (20 μg of n-heptadecanoic acid), the sample was centrifuged to deproteinize by adding 1,000 μl of ethanol, then the supernatant was evaporated. The residue was completely dried under a nitrogen stream for 3 min and derivatized with 100 μl of N, O-bis(trimetnylsilily) trifluorcetamide and 10 μl of trimethylchlorosilane at 90°C for 40 min (Zhang et al., 2000). A GCMS-QP2010 Ultra GC-MS system (Shimadzu, Kyoto, Japan) was used with an ultra Alloy capillary column (30 × 0.25 mm internal diameter with 0.25-μm film thickness, Frontier Lab, Fukushima, Japan). The temperature was programmed to increase from 60 to 350°C at 17°C/min. Next, 2 μl of derivated sample was injected in split mode. The mass chromatographic quantitation of urine metabolites including 23DH2MB was based on the fragment ion peaks area compared with the corresponding urine creatinine fragment ion area ratio.

Through retrospective re-analysis, quantitative urine screening for S-(2-caboxypropyl) cysteamine (SCPCM) was measured by LCMS/MS in urine of cases. This project was not routinely performed in the metabolic laboratory. The process included sample and label pretreatment. The SCPCM concentration in urine of 140 control children was determined by the detection and verification of an SCPCM standard, and the reference value was obtained using the SCPCM standard concentration curve.

### Data Collection and Follow-Up

At the first visit, clinical data were collected retrospectively, consisting of demographic features, early developmental milestones before first symptoms, age at presentation, initial present, precipitating causes, metabolite findings, and radiographic data.

During the follow-up period, all eight patients were assessed every 6 months to 1 year at our neurology clinic. Each follow-up evaluated motor, cognition and linguistics functions; muscle strength and tone; feeding situation; growth index; recurrence; metabolite analyses; and response to treatment.

Follow-up MRI results were classified into four levels: progression, stable, evolution, and regression (Bonfante et al., 2016). The Newcastle Pediatric Mitochondrial Disease Scale (NPMDS) was adopted to assess disease progression (Phoenix et al., 2006), and each patient was scored with the NPMDS before medication (peak period) and after treatment (the last follow-up). NPMDS scores were correlated with the degree of disease burden. Higher and lower scores indicate a more severe phenotype and clinical improvement, respectively.

### Review 34 Reported Cases With *HIBCH* Mutations

We reviewed the data of 34 published patients, of which 4 cases had insufficient clinical data (Charng et al., 2016; 336; Ceyhan-Birsoy, et al., 2019; Jou et al., 2019; Hu et al., 2020). The data of onset and current age, clinical symptoms, metabolites, neuroimaging, genotype, and natural history were analyzed to summarizes the similarities and differences between our patients and other reported cases, both with the *HIBCH* mutations.

### Analysis of Other 173 Patients With Leigh/Leigh-Like Syndrome

We grouped confirmed genes of 181 patients with Leigh/Leigh-like syndrome according to pathomechanism including OXPHOS complexes (I, II, IV, V), mitochondrial DNA-related protein synthesis, mitochondrial homeostasis, substrate, mitochondrial cofactors, and inhibitors. The age of onset, predisposing factors, initial symptoms, main symptoms, brain imaging, laboratory examinations, and clinical outcomes of 173 patients were investigated to analyze similarities and differences between subjects with *HIBCH* gene mutations and other gene mutations associated with Leigh/Leigh-like syndrome.

## Results

### *HIBCH* Genetic Analyses

**FIGURE 2:**
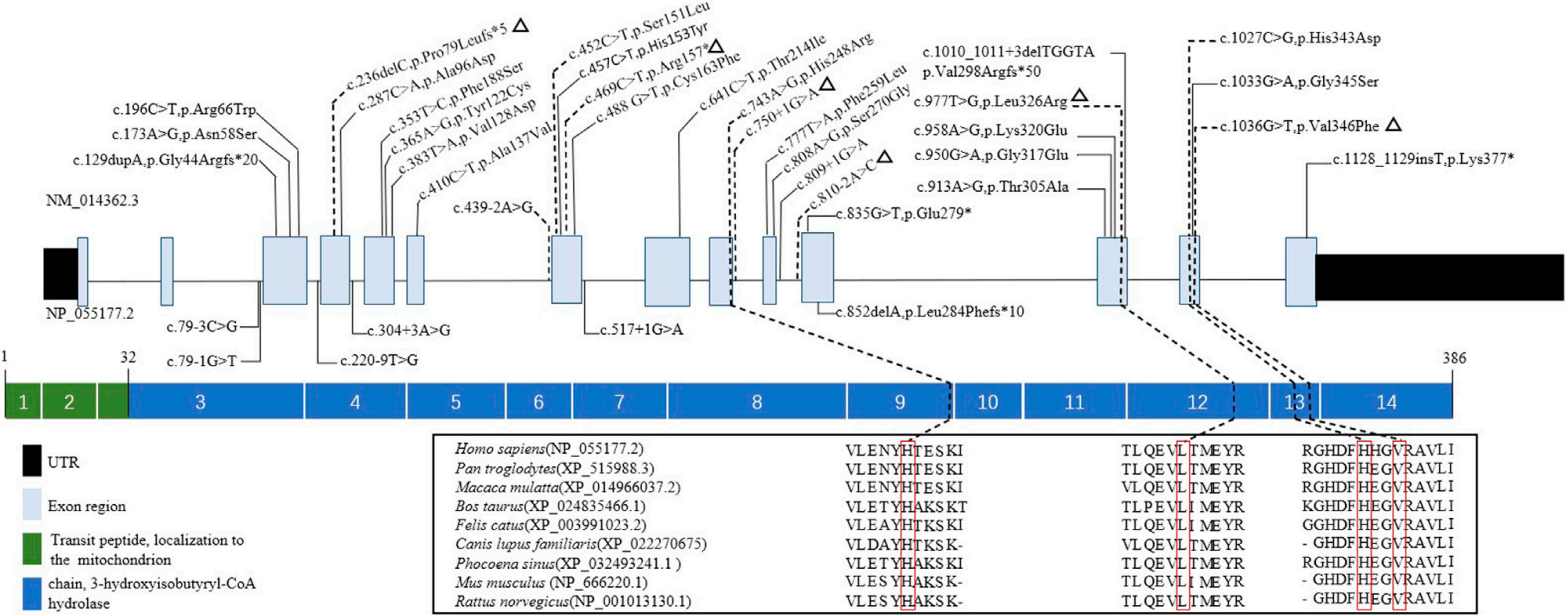
Schematic representation the *HIBCH* gene structure showing disease-associated mutation locations and conservation of mutant amino acid residues with known protein domains. Notes: dashed line, mutations in our study; triangles, our novel mutations including missense, splicing, frameshift and nonsense mutations; solid line, mutations reported in the literature.

Ten mutations, including six novel mutations, were identified by NGS of the *HIBCH* gene with compound heterozygous states (Table 1), and according to the ACMG criteria, the ten variants were classified as likely pathogenic or pathogenic (Supplementary Table S1). No other candidate genes that could fully explain clinical symptoms were found. They were confirmed by Sanger sequencing. The paternal blood samples of Patient 1 and two siblings (Patients 3 and 4) were not available. The identified mutations were distributed on coding exons 4, 7, 9, 12, and 13 (Figure 2). Two novel missense mutations, c.977T>G (p.Leu326Arg) and c.1036G>T (p.Val346Phe), were absent from the Exome Aggregation Consortium (ExAC), 1,000 Genomes, gnomAD_genome, and ESP6500 databases, but conserved amino acid residues in multiple species and several in silico tools predicted these mutations as disease causing. Two novel splicing mutations (c.750+1G>A and c.810-2A>C) were predicted as likely pathogenic. One novel nonsense mutation c.469C>T (p.Arg157*) can disrupt protein function by changing the stop codon and was also absent from population databases (gnomAD_genome, ExAC, 1,000 Genomes, and ESP6500). Another novel mutation c.236delC, p.Pro79Leufs*5, was predicted to be loss of function due to a premature truncation. The HGMD includes c.1027C>G (p.His343Asp) and c.439-2A>G, which have been reported in the literature (Zhu et al., 2015; Yang et al., 2018; Xu et al., 2019). The former was identified in four patients of this cohort, and from our NGS data there were no region of homology of the variant site. c.743A>G (p.His248Arg) and c.452C>T (p.Ser151Leu) are included in Clinvar, and both have conserved residues predicted to be damaging and disease causing. In addition, Sanger sequencing and droplet digital PCR identified maternal c.1027C>G (p.His343Asp) and a paternal 868-kb heterozygous deletion from 2q32.2 including the *HIBCH* gene in Patient 5 homozygous for the c.1027C>G (p.His343Asp) *HIBCH* variant. Our identified 6 novel mutation and 31 other reported mutation sites are summarized in Figure 2.

**TABLE 1:** *HIBCH* mutations in eight patients.

Patient	Exon	Nucleotide variation^a^	Amino acid variation^b^	Reported/novel	Parental origin	AF	Predication of pathogenicity			
							Mutation taster (score)	SIFT (score)	CADD (score)	PROVEAN (score)
**1**	12	c.977T>G	p.Leu326Arg	Novel	ND	—	Disease causing (1)	Damaging (0.00)	28.1	Damaging (−5.12)
	13	c.1027C>G	p.His343Asp	Reported	Maternal	4.067e-06	Disease causing (1)	Tolerable (0.089)	22.9	Damaging (−3.88)
**2**	7	c.452C>T	p.Ser151Leu	Reported	Maternal	—	Disease causing (1)	Damaging (0.0)	34.0	Damaging (−5.79)
	7	c.469C>T	p.Arg157*	Novel	Paternal	1.22e-05	Disease causing (1)	NA	43.0	NA
**3**		c.750+1G>A	NA	Novel	Maternal	—	NA	NA	34.0	NA
	13	c.1036G>T	p.Val346Phe	Novel	ND	—	Disease causing (1)	Damaging (0.00)	35.0	Damaging (−4.03)
**4**		c.750+1G>A	NA	Novel	Maternal	—	NA	NA	34.0	NA
	13	c.1036G>T	p.Val346Phe	Novel	ND	—	Disease causing (1)	Damaging (0.00)	35.0	Damaging (−4.03)
**5**	13	c.1027C>G	p.His343Asp	Reported	Maternal	4.067e-06	Disease causing (1)	Tolerable (0.089)	22.9	Damaging (−3.88)
		868 kb deletion including *HIBCH*		Novel	Paternal	NA	NA	NA	NA	NA
**6**	13	c.1027C>G	p.His343Asp	Reported	Paternal	4.067e-06	Disease causing (1)	Tolerable (0.089)	22.9	Damaging (−3.88)
		c.439-2A>G	NA	Reported	Maternal	—	NA	NA	34.0	NA
**7**	4	c.236delC	p.Pro79Leufs*5	Novel	Paternal	—	Disease causing (1)	NA	NA	NA
	13	c.1027C>G	p.His343Asp	Reported	Maternal	4.067e-06	Disease causing (1)	Tolerable (0.089)	22.9	Damaging (−3.88)
**8**		c.810-2A>C	NA	Novel	Maternal	—	NA	NA	34.0	NA
	9	c.743A>G	p.His248Arg	Reported	Paternal	8.86e-06	Disease causing (1)	Damaging (0.03)	23.1	Damaging (−3.37)

^a^ Transcript, NM_014362.3; Allele Frequency; —, Unrecorded; ND, not detection; NA, not available.

^b^ NP_055177.2.

### Metabolite Results and Laboratory Investigations

**FIGURE 3:**
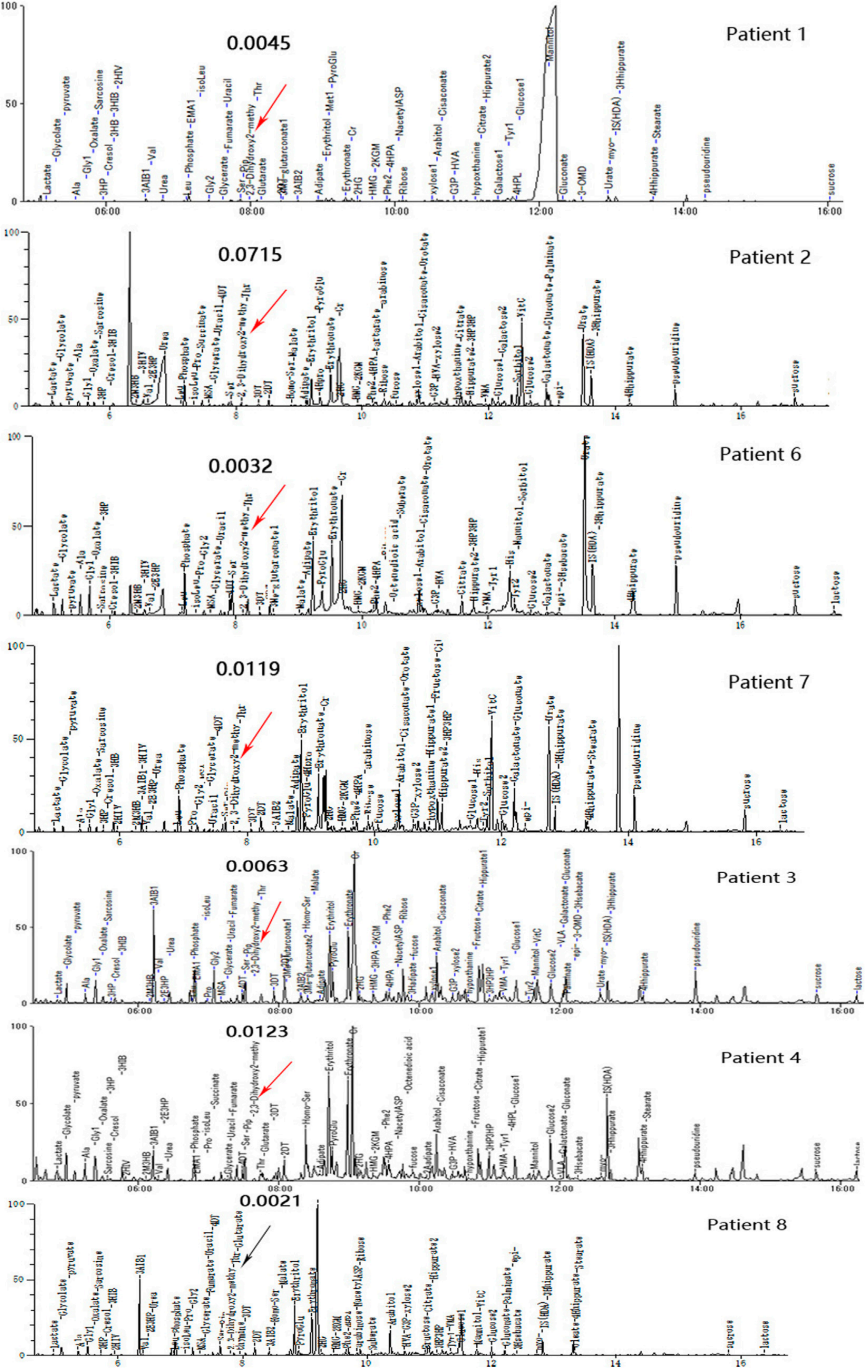
Urine organic acid profiles of seven patients in different phases; red arrows indicate abnormally high 23DH2MB peaks, and the black arrow represents a normal 23DH2MB peak. Patients 1, 2, 6, and 7 with elevated 23DH2MB levels in the peak stage. Patients 3 and 4 with elevated 23DH2MB levels in the recovery stage. Patient 8 with a normal 23DH2MB level after adopting a valine-restricted diet.

There were 5 (5/8) patients with DBS showing elevated C4-OH during either the peak or recovery phase. Six (6/7) had increased 23DH2MB levels in urine (Figure 3), including four cases in the peak stage and two in the recovery stage. Through retrospective analysis, we identified an increase in urine SCPCM levels in three cases. The data are shown in Table 2. There were no significant abnormalities of amino acid levels in DBS. In addition, three patients had mild elevation of lactic acid in blood, but no cases had elevated lactate in cerebrospinal fluid.

**TABLE 2:** Metabolite results of extensive investigations of eight patients with *HIBCH* mutations.

	C4-OH (0.00–0.26 μmol/L)			23DH2MB (0.0005–0.0029)		SCPCM (<0.624 μmol/mmol Cr)		Lactic acid	
	Peak phase	Recovery phase	Neonatal period	Peak phase	Recovery phase	Peak phase	Recovery phase	Blood (0.5–2.2 mmol/L)	CSF (1.0–2.78 mmol/L)
Patient 1	0.200	—	—	0.0045↑	—	—	—	1.51	1.46
Patient 2	1.664↑	—	—	0.0715↑	—	8.02↑	—	4.13↑	—
Patient 3	—	0.184	—	—	0.0063↑	—	—	1.60	—
Patient 4	0.579↑	0.183	—	—	0.0123↑	—	2.74↑	0.92	—
Patient 5	0.221	—	—	—	—	—	—	5.23↑	—
Patient 6	0.73↑	0.174	—	0.0032↑	—	2.45↑	—	3.23↑	—
Patient 7	0.485↑	—	—	0.0199↑	—	—	—	1.50	—
Patient 8	1.574↑	1.235↑	0.425↑	—	0.0021	—	—	1.2–2.1	1.50

↑, elevated level; —, not detected; CSF, cerebrospinal fluid.

Additionally, we found that the NPMDS scores during the peak phase may be correlated with the value of C4-OH and SCPCM. We also observed that patients with lower C4-OH during the peak phase were more likely to have a positive response to treatment.

### Demographic and Clinical Features

**FIGURE 4:**
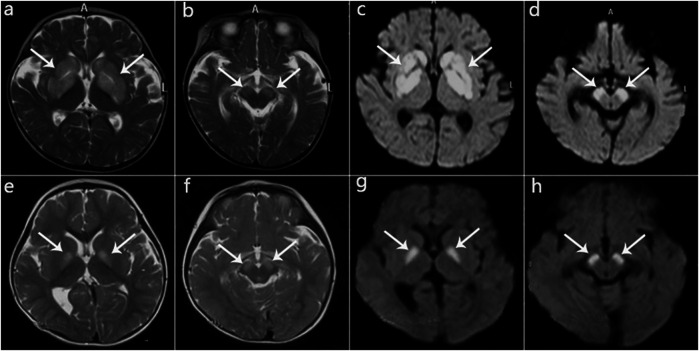
MRI manifestations of 2 patients (Patient 2, Patient 5) during disease onset. **(a‐d)**: Patient 2 at age 10 months; hyperintensity of the globus pallidus, putamen, caudate nucleus, and cerebral peduncle on T2WI **(a,b)** and DWI **(c,d)**. **(e‐h)**: Patient 5 at age 13 months; hyperintensity on T2WI **(e,f)** and DWI **(g,h)** in the globus pallidus and cerebral peduncle.

In this case series, eight patients (four male, four female) were from seven non-consanguineous Chinese families, and Patients three and four were siblings. The clinical features are summarized in Table 3. Two patients were ethnic minorities (Patient 6 Mongolian and Patient 8 Hmong). Seven were born at term with normal birth measurements, and the perinatal period was generally normal for all subjects. Early developmental milestones before disease onset were almost normal. All patients developed initial symptoms at ages ranging from 8 to 18 months at a median of 13 months. With regard to the initial symptoms, three cases developed encephalopathy, two cases had developmental regression, two cases showed developmental delay, and one case was dystonia. Six patients had precipitating causes, with five cases triggered by infection and one case by vaccination. Brain MRI scans of all cases at onset revealed T2 hyperintensity in the bilateral basal ganglia, and four cases also had brainstem lesions, similar to manifestation. In short, it was Leigh pattern imaging (Figure 4).

**TABLE 3:** Clinical features and follow-ups of eight patients with *HIBCH* mutation.

	Patient 1	Patient 2	Patient 3	Patient 4	Patient 5	Patient 6	Patient 7	Patient 8
Gender	Female	Female	Male	Male	Female	Male	Female	Male
Ethnic origin	Han	Han	Han	Han	Han	Mongolian	Han	Hmong
Perinatal problems	—	—	—	—	DFM	Jaundice	—	—
Age at onset	1 years 2 months	10 months	1 year 6 months	8 months	1 year 1 months	1 year 8 months	1 year 1 months	11 months
Initial presentation	DR	Encephalopathy	DD	Encephalopathy	DR	PD	DD	Encephalopathy
Precipitating causes	Pneumonia	Diarrhea	—	Febrile illness	ES	HAV	—	Influenza
Age at diagnosis	4 years 7 months	1 year	5 years 8 months	4 years	1 year 2 months	2 years 2 months	1 year 10 months	1 year 2 months
Developmental regression/delay	+/+	+/+	+/+	+/+	+/+	+/+	−/+	+/+
Hypotonia	+	+	+	+	+	+	+	+
Encephalopathy	+	+	+	+	−	+	−	+
Metabolic acidosis	−	+	−	+	−	+	−	+
Feeding difficulties	−	+	+	+	−	+	−	+
Dystonia	−	+	+	−	−	+	−	+
Ataxia	+	−	+	+	−	−	−	+
Strabismus	−	−	+	+	−	+	−	+
Nystagmus	−	−	+	+	−	−	−	+
Seizures	−	−	+	+	−	−	−	+
MRI-basal ganglia of T2 hyperintensity	+	+	+	+	+	+	+	+
MRS (Lactate peak)	−	ND	+	−	ND	−	ND	−
NMPDS score in the peak phase	35.4	45.8	36.2	44.8	38.1	45.6	34.2	48.8
Therapy	+^a^	−	−	−	+^a^	+^b^	+^b^	+^b^
Age of last assessment	8 years 4 months	4 years	8 years	6 years 4 months	2 years 4 months	3 years 2 months	2 years 7 months	2 years 3 months
Follow-up MRI	Evolution	NA	Progression	Progression	NA	Stable	Evolution	Progression
NMPDS score in the last assessment	5.2	61.3	53.1	48.4	19.9	32.1	15.8	38.7

DFM, decreased fetal movement; DR, developmental regression; DD, developmental delay; PD, paroxysmal dystonia; ES, exanthema subitum; HAV, hepatitis A vaccine; ND, not detection; NA, not available.

^a^ Therapy with drug [L‐carnitine (50 mg/kg/day), coenzyme Q10 (10 mg/kg/day), thiamine (10 mg/kg/day), riboflavin (10 mg/kg/day) and symptomatic drugs].

^b^ Therapy with drug plus valine-restricted diet; Progression, new lesions present and/or extension of previously visualized lesions; Stable, no change in T2 or DWI; Evolution, normalization of DWI with persistent T2 changes, or decreased size of the T2 signal changes as a result of encephalomalacia.

The median age of diagnosis was 2.0 years (range 1.0–5.7 years). During the disease course, the frequently prominent clinical features were hypotonia (8/8), developmental delay (8/8), developmental regression (7/8), encephalopathy (6/8), feeding difficulties (5/8), dystonia (4/8), ataxia (4/8), metabolic acidosis (4/8), strabismus (4/8), nystagmus (3/8), and seizures (3/8). Video electroencephalogram (VEEG) in two cases (Patients 3 and 4) with tonic seizures showed bilateral central and apical areas with sharp and slow wave emission that was more remarkable on the right side. The VEEG of Patient 8 with focal seizures presented as continuous release of slow waves and multiple spikes in the right frontal and temporal regions. Two cases (Patients 3 and 8) developed status epilepticus during acute encephalopathy. Three (Patients 2, 3, and 8) developed respiratory failure during acute encephalopathy, and all required intubation and ventilatory support for ∼1 week, of which one case (Patient 3) had a failed extubation and underwent a second intubation for ventilator-assisted breathing. Two cases (Patients 2 and 8) had two episodes of acute encephalopathy both induced by infection in 1 year. During acute encephalopathy period, two cases (Patient 2 and 4) were both associated with thyroid dysfunction (T3, T4 and TSH all decreased) and one case (Patient 6) with hepatic dysfunction (ALT 375 U/L, reference range 0–40 U/L), and about half a year after the peak period, these indicators gradually returned to normal.

### Treatment

Once the patients diagnosed, we fully informed the parents about treatments and recommended pharmacologic therapy and adopting a valine-restricted diet. The drugs consisted of antioxidants and OXPHOS complex cofactors including L-carnitine (50 mg/kg/day), coenzyme Q10 (10 mg/kg/day), thiamine (10 mg/kg/day), and riboflavin (10 mg/kg/day), additionally including some symptomatic drugs, such as levetiracetam, or baclofen. For the dietary treatment, we worked with nutritionists to develop a plan to limit valine intake with protein intake of 1.0–1.5 g/kg/d, recommended energy intake of 80 kcal/kg/d, and no restriction on carbohydrates. It was recommended to use maple glycosuria formula milk powder and supplement leucine and isoleucine.

**FIGURE 5:**
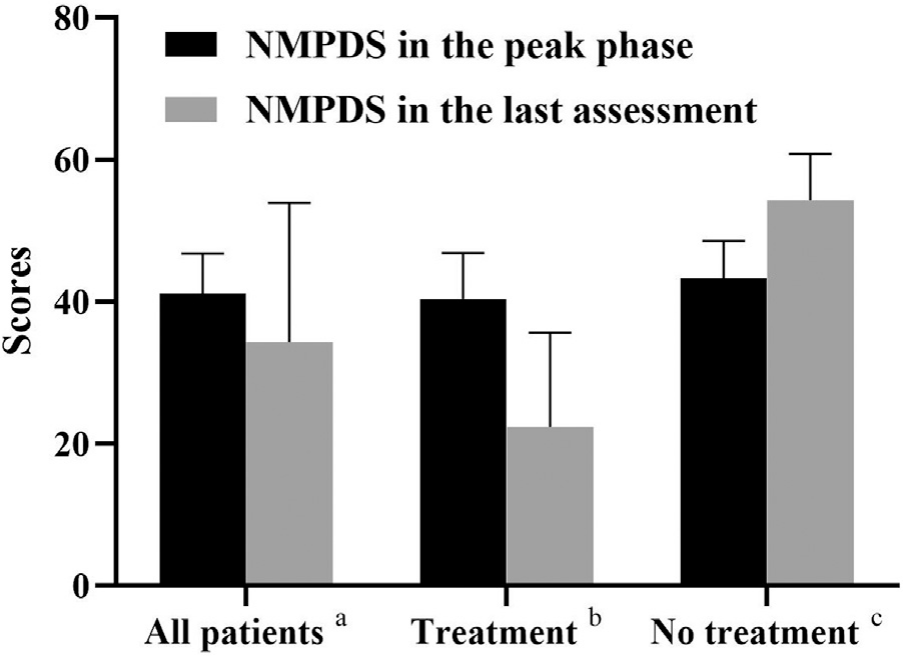
NMPDS scores in the peak phase and last assessment. a, All the recruited patients (n = 8); b, Patients who received therapy (Patient 1,5,6,7,8; n = 5); c, Patients who gave up therapy (Patient 2,3,4; n = 3).

Two cases (Patients 1 and 5) only used drug treatments, 3 (Patients 6, 7, and 8) initiated drug and diet therapy, and 3 cases (Patients 2, 3, and 4) abandoned therapy. The NPMDS scores in the peak phase and at the last follow-up (recovery phase) were shown in Figure 5 **.** The treated patients presented with significant decreased scores.

### Follow-Up

The median duration between initial symptom onset and the last follow-up was 2.3 years (range 1.3–7.2 years). NPMDS scores during the peak phase and at the last evaluation, as well as MRI findings were showed in Table 3. The clinical conditions of three cases (Patients 1, 5, and 7) improved, while 2 (Patient 6 mild delay and Patient 8) were relatively stable; all five cases were in the treatment group. Conversely, all three cases (Patients 2, 3, and 4) in the non-treatment group progressed.

**FIGURE 6:**
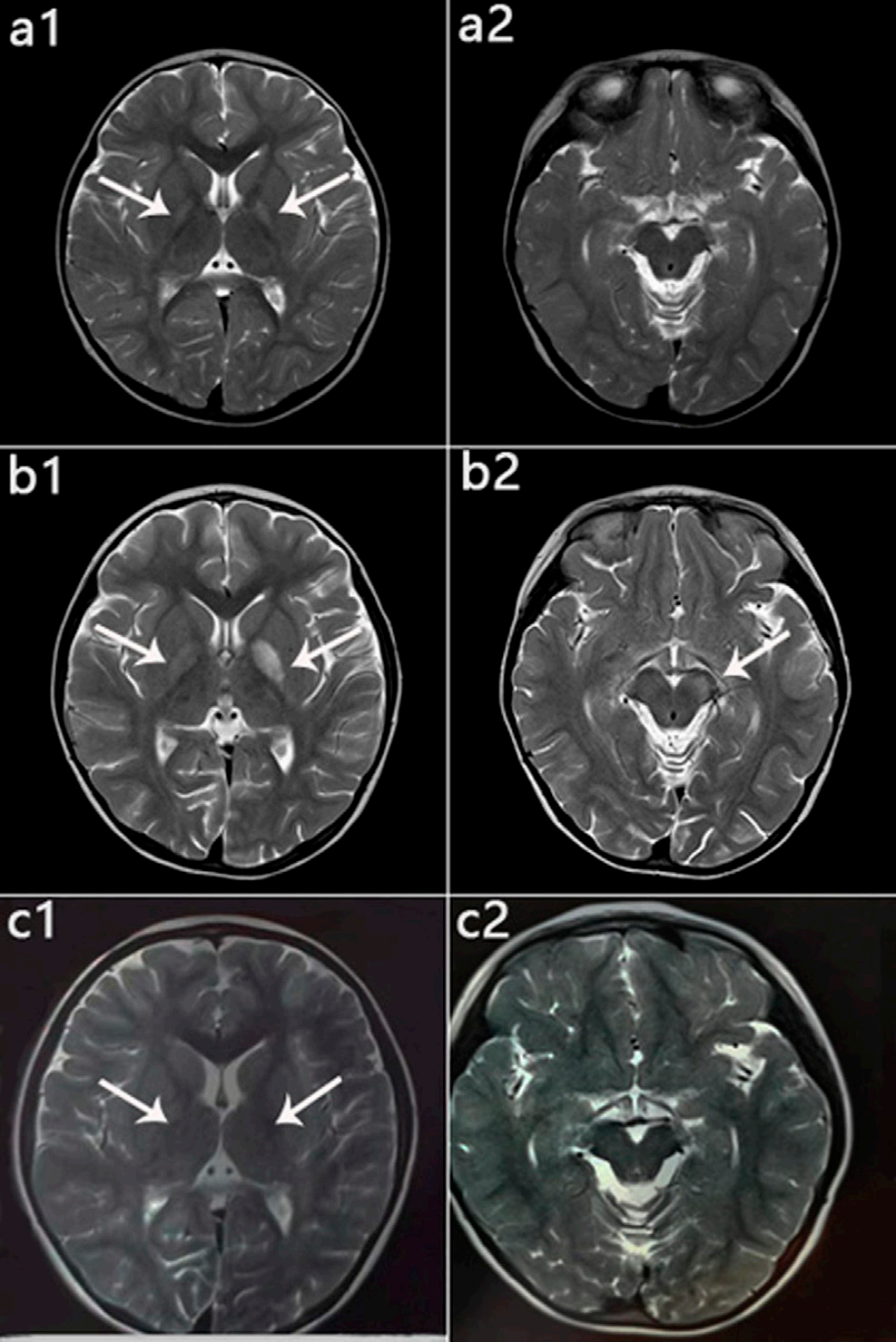
Patient 1: MRI performance in the peak and recovery phases. **(a1,a2)**: Initial MRI obtained at age 2 years; hyperintensity on T2WI in the globus pallidus **(a1)**, normal cerebral peduncle **(a2)**. **(b1,b2)**: MRI performed at 4 years 5 months during the acute stage; hyperintensity on T2WI in the globus pallidus with swelling on the left side **(b1)** and in the left cerebral peduncle **(b2)**. **(c1,c2)**: MRI performed at 6 years 1 month during the recovery stage; only slightly hyperintensity on T2WI in globus pallidus **(c1)**, and Abnormal signal disappeared in the cerebral peduncle **(c2)**.

Patient 1 exhibited developmental regression following pneumonia at age 14 months but subsequently recovered. Unfortunately, at 4 years 5 months, she experienced acute encephalopathy due to a febrile infection, which again led to developmental regression, and she lost the skills of sitting and speaking. She received drug therapy thereafter and slowly recovered and gradually improved. The patient is currently 8 years 4 months old, with a normal growth index. She is independent and attends grade 3 at a normal elementary school but sometimes has trouble concentrating and wandering. She has some movement intolerance and walks with a slightly abnormal right lower limb posture. Despite having several fevers and other infections since initiating treatment, none have worsened her condition. Her last MRI scan showed significant improvement (Figure 6).

For Patient 5, roseola infantum at age 13 months caused her condition to deteriorate, presenting with no head control, no language, and unable to sit unassisted. Following drug therapy, she gradually recovered. Now she is 2 years 4 months old, can understand basic sentences, walk a dozen steps unsupported, and speak 2 and 3 words.

Patient 7 has received drug and diet treatments for 6 months and has improved. The therapy included drugs of L-carnitine 500 mg/day, coenzyme Q10 100 mg/day, thiamine 100 mg/day, riboflavin 100 mg/kg/day, and limit protein intake 12 g/d, no restriction on carbohydrates. At present she is 2 years 7 months, can walk without assistance but occasionally falls, can understand complex sentences, and pronounce monosyllables.

Patient 6 have received the standard therapy of drug and diet plus baclofen (1 mg/kg/day) since the onset of encephalopathy for nearly 1 year. Despite four bouts of pneumonia during this time, his condition did not deteriorate. He is 3 years and 2 months old, with dystonia as the main manifestation. He can walk for a few steps with support, understand general language, and pronounce sequential words.

Patient 8 was initiated on drug therapy plus levetiracetam (30 mg/kg/day) since onset at age 11 months with partial responses. However, 10 months later he experienced a second encephalopathy episode followed by frequent seizures and status epilepticus. Since being placed on a valine-restricted diet, he has shown a dramatic response. Although he has been seizure-free for 5 months, there has been no improvement in development. He is 2 years 4 months old, with no head control, language, or eye contact.

The conditions of all three cases without long-term treatment (Patients 2, 3, and 4) deteriorated. They had poor response to treatment in the early stage, and all eventually stopped therapy. At the last follow-up, Patient 2 was 4 years old, with height 90 cm (<3rd percentile) and weight 8.5 kg (<3rd percentile). She has poor head control with no eye contact or language. Patient 3 is currently 8 years old. He has no head control and is unable to sit unsupported or actively grasp objects. He knows simple words but is nonverbal. Patient 4 is presently 6 years 4 months old; his clinical course and physical examination are similar to that of his older brother (Patient 3).

Six (6/8) patients underwent follow-up neuroimaging (Table 3). Brain MRI showed progression in three cases, two of which abandoned therapy, and 1 (Patient 8) case with therapy had lesion progression to the entire right cerebral hemisphere with evident brain volume loss. One case (Patient 3) had corpus callosum thinning and a lactic acid peak on magnetic resonance spectroscopy (MRS). Brain MRI improved in two cases and was relatively stable in one case, and they were all receiving therapy.

### Analysis of 34 Reported and Our Cases With *HIBCH* Mutations

The patient's general condition, clinical, imaging, and metabolic characteristics were summarized in Table 4, and there were no significant statistical difference between our cases series and other reported cases. In our cohort, there were no deaths, probably due to the older age of onset, but 7 (22%) of the reported cases had been fatal. The main symptoms of total cases were developmental delay/regression, hypotonia, distonia, encephalopathy, and feeding difficulties. T2 hyperintensity in the bilateral basal ganglia occurs in an average of 95% of cases. Brain atrophy and brainstem lesions were also common neuroimaging abnormalities. All patients tested for SCPCM presented with elevated levels, suggesting a more specific metabolite of the disease, and more samples were needed for verification. In both groups, there was an almost equal proportion (63 vs. 65%) of cases with elevated C4-OH levels. However, the percentage of patients with elevated 23DH2MB appeared to be higher in our cases, but the difference was not statistically significant due to the small sample size.

**TABLE 4:** The clinical, neuroimaging and metabolic characteristics of our cases and the reported *HIBCH* mutation patients.

	Our cases	Reported cases	Total cases
General demographic feature**s**	N = 8	N = 32	N = 40
Sex	4 F: 4 M	17 F: 15 M	21 F: 19 M
Family history	2 (25%)	15 (47%)	17 (43%)
Age onset, median (range)	13, (8–20) months	4, (0–84) months	6, (0–84) months
Precipitating cause	6 (75%)	12 (38%)	18 (45%)
Current age, median (range)	4.0, (2.3–8.3) years	5.0, (0.25–43) years	5.0, (0.25–43) years
Death cases	0	7 (22%)	7 (18%)
Death age, median (range)	—	36, (0.9–96) months	36, (0.9–96) months
Main clinical features	N = 8	N = 30	N = 38
Developmental delay	5 (63%)	24 (80%)	29 (76%)
Developmental regression	7 (88%)	17 (57%)	24 (63%)
Hypotonia	8 (100%)	20 (67%)	28 (74%)
Encephalopathy	6 (75%)	13 (43%)	19 (50%)
Dystonia	4 (50%)	15 (50%)	19 (50%)
Feeding difficulties	5 (63%)	14 (47%)	19 (50%)
Ataxia	4 (50%)	11 (37%)	15 (40%)
Seizures	3 (38%)	10 (33%)	13 (34%)
Nystagmus	3 (38%)	11 (37%)	14 (37%)
Strabismus	4 (50%)	6 (20%)	10 (26%)
Optic atrophy	0	5 (17%)	5 (13%)
Brain imaging involvement**s**	N = 8	N = 30	N = 38
Basal ganglia	8 (100%)	28 (93%)	36 (95%)
Brainstem	6 (75%)	15 (50%)	21 (55%)
White matter	3 (38%)	10 (33%)	13 (34%)
Cerebellum	3 (38%)	7 (23%)	10 (26%)
Brain atrophy	3 (38%)	18 (60%)	21 (55%)
Corpus callosum	1 (13%)	5 (17%)	6 (16%)
MRS lactate peak	1 (13%)	3 (10%)	4 (11%)
Metabolic studies			
Increased blood lactate level	3/8 (38%)	12/27 (44%)	15/35 (43%)
Elevated urine SCPCM	3/3 (100%)	2/2 (100%)	5/5 (100%)
Elevated blood C4-OH	5/8 (63%)	15/23 (65%)	20/31 (65%)
Elevated urine 23HD2MB	6/7 (86%)	2/8 (25%)	8/15 (53%)
Decreased HIBCH activity in skin fibroblasts	—	11/11 (100%)	11/11 (100%)

### Analysis With 173 Patients With Leigh/Leigh-Like Syndrome

There were 43 other mutant genes identified in 173 cases with Leigh/Leigh-like syndrome (Supplementary Figure S1). We summa**r**ized the clinical, genetic, metabolic and imaging characteristics of the 173 cases. Due to baseline heterogeneity and large differences in sample size, we only performed statistical descriptions of the data (Supplementary Table S2). The symptoms of hypotonia, dystonia, encephalopathy, and feeding difficulties were more common in patients with *HIBCH* mutations. In metabolites, the elevated levels of blood C4-OH (62.5 vs. 2.3%) and urine 23DH2MB (85.7 vs. 5.8%) were significant in the *HIBCH* mutation group. Among the 173 patients group, 10 cases showed elevated 23DH2MB levels, including 8 with *ECHS1* mutation, 1 with *SURF1* mutation, and 1 with *MT-ND6* mutation, and the latter 2 patients showed slightly elevated 23DH2MB levels and normal C4-OH levels. This implied that elevated levels of C4-OH combined with 23DH2MB in patients with clinically diagnosed Leigh/Leigh-like Syndrome may serve as specific metabolites for *HIBCH* mutation patients.

## Discussion

We analyzed the clinical, metabolic, and genetic features of 8 patients with *HIBCH* mutations associated with Leigh/Leigh-like syndrome and reported their follow-up data providing additional natural history information. We also investigated the relationships between metabolite levels, disease severity, and clinical outcomes.

In our case series, earlier onset age was associated with a poorer prognosis. Infection or vaccination could also accelerate or exacerbate the onset or recurrence. There have been 34 patients from 27 unrelated families reported with *HIBCH* mutations, 4 of which have insufficient clinical data. Twenty-six (26/32) had initial symptoms within 2 years of age, including seven cases who died within an onset age of 1 year old (Brown et al., 1982; Ferdinandusse et al., 2013; Yamada et al., 2014; Peters et al., 2015; D'Gama et al., 2020). The significant clinical presentations were developmental delay/regression, hypotonia, encephalopathy, feeding difficulties and dystonia, and the last 4 symptoms were more specific in patients with Leigh/Leigh-like syndrome caused by *HIBCH* mutation in our cohort. Interestingly, seizures seemed to be related to phenotype severity, but it occurred infrequently with only 3 patients in this cohort. Among 10 (10/33) patients described elsewhere, most of had poor responses to treatment and worse prognoses (Loupatty et al., 2007; Ferdinandusse et al., 2013; Reuter et al., 2014; Candelo et al., 2019; Karimzadeh et al., 2019; D'Gama et al., 2020; Marti-Sanchez et al., 2020). The neuroimaging of all 8 patients showed symmetrical lesions in basal ganglia with/without brainstem. Lactate peaks on MRS (Soler-Alfonso et al., 2015; Candelo et al., 2019; D'Gama et al., 2020) and corpus callosum abnormalities (Brown et al., 1982; Peters et al., 2015; Tan et al., 2018; Candelo et al., 2019; D'Gama et al., 2020) are uncommon in cases with *HIBCH* mutation, but it may be associated with a more severe phenotype. Additionally, in our cohort there were 2 cases with thyroid dysfunction during the onset phase who had negative prognoses, and only 1 case were reported in the recent literature (Marti-Sanchez et al., 2020). Furthermore, one case had hepatic dysfunction as a rare presentation, and it was proposed that HIBCH might protect cells against methacrylyl-CoA toxicity (Ishigure et al., 2001). The follow-ups showed differences in the prognoses of these patients, which may be related to therapeutic interventions or their genotype.

It is difficult to correlate the genotypes and phenotypes of HIBCH deficiency. The variation sites reported to date are distributed in all exons except exons 1 and 2. Of these, exon 12 has the most mutation sites and cases, but phenotype severity varies (Ferdinandusse et al., 2013; Schottmann et al., 2016). Additionally, there have been no identified pathogenic mutations impacting the key residues of Glu120, Gly146, Glu169, or Asp177 (substrate binding). It was reported that truncating mutations tend to cause a more severe phenotype (Tan et al., 2018). One study observed a longer survival in cases with homozygous mutations located on the protein surface than in those with variants inside or near the catalytic site (Marti-Sanchez et al., 2020). Disease severity may be related to residual HIBCH enzyme activity (Yamada et al., 2014). The p.His343Asp mutation seemed to be associated with less severe clinical presentations in this cohort. To date, there has been no confirmed founder or hot spot mutation of the *HIBCH* gene. In our cases, c.1027C>G [p.His343Asp] was identified in half of patients, in addition to two of the four Chinese cases previously reported (Zhu et al., 2015; Xu et al., 2019), but it has not been described in other countries. Additionally, there is no region of homology from our NGS data, and it is at a extremely low frequency in population databases. We speculate that p.His343Asp may be a hot spot mutation in Chinese. The major limitation of our study is the absent detection of HIBCH enzyme activity in recruited patients. Instead, we assessed specific metabolites in blood and urine, so that patients could easily accept and assist in disease diagnosis.

HIBCH deficiency leads to the accumulation of 3-hydroxy-isobutyryl-CoA, but it was hypothesized that methacrylyl-CoA (Figure 1), as an upstream highly reactive intermediates, resulted in disease pathogenesis and neurotoxicity. The excessive generated methacrylyl-CoA can react with cysteamine to form SCPCM. 23DH2MB was derived from acryloyl-CoA, a compound homologous to methacrylyl-CoA, involving in valine metabolic pathway. In our cohort, elevated C4-OH and 23DH2MB levels are rare in Leigh/Leigh-like syndrome patients with other gene mutations. Our results indicate that C4-OH in the blood and 23DH2MB and SCPCM in the urine can be used as specific metabolites to assist the diagnosis and evaluate disease severity, although they are not completely specific.

The elevated C4-OH level is the direct result of the accumulation of 3-hydroxy-isobutyryl-CoA, according to acylcarnitine analyses in approximately 15 reported patients with confirmed *HIBCH* mutations. In this cohort, a higher C4-OH level in the peak phase was associated with a more severe phenotype, indicating poor prognosis. However, in current clinical laboratories, typical LCMS/MS cannot distinguish between 3-hydroxyisobutyryl-carnitine and 3-hydroxybutyryl carnitine isomers. This requires comprehensive clinical analysis. Sometimes C4-OH was partially elevated in the asymptomatic neonatal period (Stiles et al., 2015) and was easily overlooked. For example, Patient 8 had a mild increase in C4-OH in the asymptomatic neonatal period, but it did not attract enough attention to make a timely diagnosis and initiate treatment.

Probably due to different stages and different severity of the disease, 23DH2MB in our cases showed a higher positive rate compared with reported cases. 23DH2MB levels did appear to change in response to treatment. After Patient 8 adopted a valine-restricted diet for 1 month, 23DH2MB fell to the reference value, while C4-OH had not yet returned to a normal level. The first measurement of 23DH2MB was in normal urine with trace amounts (Thompson et al., 1975), and also detected in patients with propionate disorders. In the valine pathway, it is originated from acryloyl-CoA, but the exact mechanism is not clear. 23DH2MB abnormalities were also identified in a few Leigh syndrome patients with *ECHS1* mutations (Peters et al., 2014; Yang and Yu, 2020). There was only 2 reported patient with an *HIBCH* mutation with elevated urinary 23DH2MB excretion (Ferdinandusse et al., 2013; Marti-Sanchez et al., 2020). We identified 6 patients with *HIBCH* mutations with elevated 23DH2MB levels.

SCPCM as a conjugate of the toxic metabolite methacrylyl-CoA, was firstly identified in HIBCH deficiency patients (Brown et al., 1982). It was an unusual amino acid in the urine of *HIBCH* mutation (Peters et al., 2015) and *ECHS1* mutation patients, and cannot be detected in routine metabolic screening. In our case series, urinary SCPCM level correlated with both disease severity and prognosis. To date, there have been 5 patients identified with *HIBCH* mutations and elevated SCPCM levels in urine, including 3 cases in our study. SCPCM seems to be more specific for disease diagnosis, but more samples are needed for verification.

There is currently no consensus on HIBCH deficiency treatment approaches. A low-valine, carbohydrate-rich diet may be effective (Loupatty et al., 2007; Soler-Alfonso et al., 2015). This treatment can avoid excessive ATP production from aberrant valine metabolism. Other possible drug therapies involve the administration of antioxidants and supplementation with vitamins/cofactors, often termed nutraceuticals. Fewer than 10 cases have reported treatment outcomes that include improvement (Loupatty et al., 2007; Soler-Alfonso et al., 2015), progression and even death (Yamada et al., 2014). It is difficult to correlate therapeutic effects with gene mutation sites and clinical manifestations, which may be related to residual HIBCH enzyme activity. Patients in this cohort with the c.1027C>G [p.His343Asp] mutation seemed to responded well to treatment, similar to previous studies (Zhu et al., 2015; Xu et al., 2019).

In real clinical work, metabolites can be affected by the severity of disease and diet state, and false positive or negative results may occur. Genetic testing, especially targeted NGS (WES/panels), is the first-line auxiliary diagnostic approach. However, the targeted NGS testing is costly, and it can not identify possible pathogenic variants in deep intron regions and larger deletions/duplications. If the metabolites and clinical phenotypes are well defined and consistent, it may be recommended to directly perform the less costly approach of Sanger sequencing. Unfortunately, we did not perform enzymatic detection of HIBCH. We should combine clinical, metabolic and genetic analysis to diagnose the disease. Early diagnosis and treatment may delay disease progression of the disease. The main limitations of our study were the small sample size and the absence of formal clinical trials.

In conclusion, we identified 10 rare *HIBCH* gene variants in 8 patients with Leigh/Leigh-like syndrome, including 6 novel variants. The phenotype of Leigh/Leigh-like syndrome caused by *HIBCH* mutations was not significantly different from other gene mutations, except for more comment presentations with hypotonia, dystonia, and acute encephalopathy. The genotype-phenotype correlation requires further research. However, measurements of metabolites including C4-OH, 23DH2MB and SCPCM were relatively specific and also associated with disease severity, therapeutic effects, and possibly prognosis. The non-invasive tools of NGS and metabolite analyses should be considered first-line auxiliary diagnostic approaches. Patients with Leigh/Leigh-like syndrome with *HIBCH* mutations may have a positive prognosis, especially with early diagnosis and timely therapy.

## Data Availability

The data that support the findings of this study are available from the corresponding author upon reasonable request.
