# Corrigendum: Cinical, Metabolic, and Genetic Analysis and Follow-Up of Eight Patients With *HIBCH* Mutations Presenting With Leigh/Leigh-Like Syndrome

**DOI:** 10.3389/fphar.2021.686933

**Published:** 2021-06-10

**Authors:** Junling Wang, Zhimei Liu, Manting Xu, Xiaodi Han, Changhong Ren, Xinying Yang, Chunhua Zhang, Fang Fang

**Affiliations:** ^1^ Department of Neurology, Beijing Children’s Hospital, Capital Medical University, National Center for Children’s Health, Beijing, China; ^2^ Department of Research, Development of MILS International, Ishikawa, Japan

**Keywords:** HIBCH gene, Leigh/Leigh-like syndrome, C4-OH, 2,3-dihydroxy-2-methylbutyrate, mitochondrial disorders, children

In the original article, there was a mistake in the legend for **Figure 3** as published. The word “23HD2MB” in the last sentence of the legend was misspelled, and it should be “23DH2MB.”

Furthermore, there was a mistake in the positions of Figures 5, 6 as published. The positions of Figures 5, 6 are misplaced, and they should be interchanged. The corrected Figures 5, 6 appear below.

**FIGURE 5:**
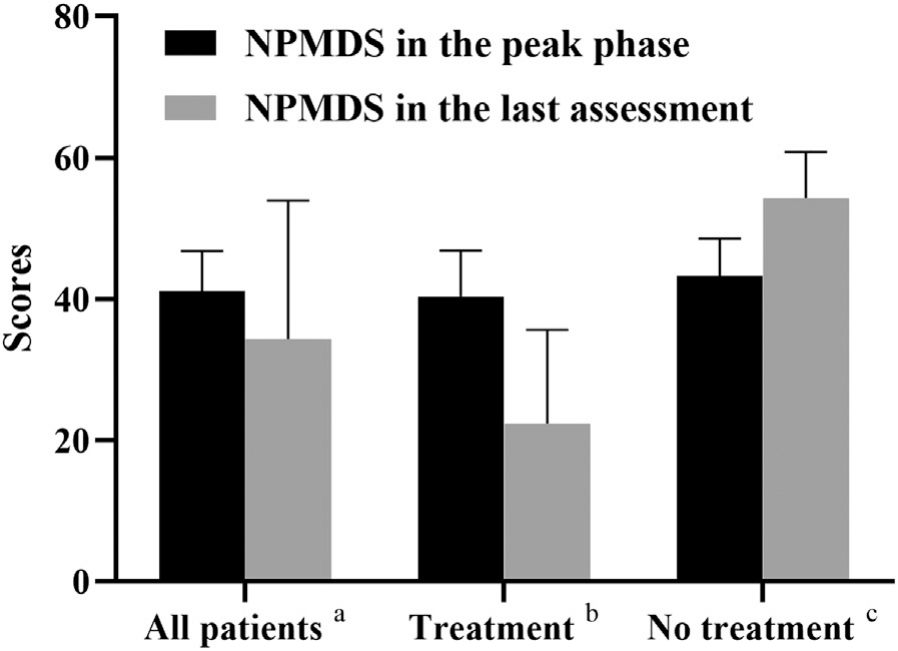
NPMDS scores in the peak phase and last assessment. a, All the recruited patients (*n* = 8); b, Patients who received therapy (Patient 1, 5, 6, 7, 8; *n* = 5); c, Patients who gave up therapy (Patient 2, 3, 4; *n* = 3).

**FIGURE 6:**
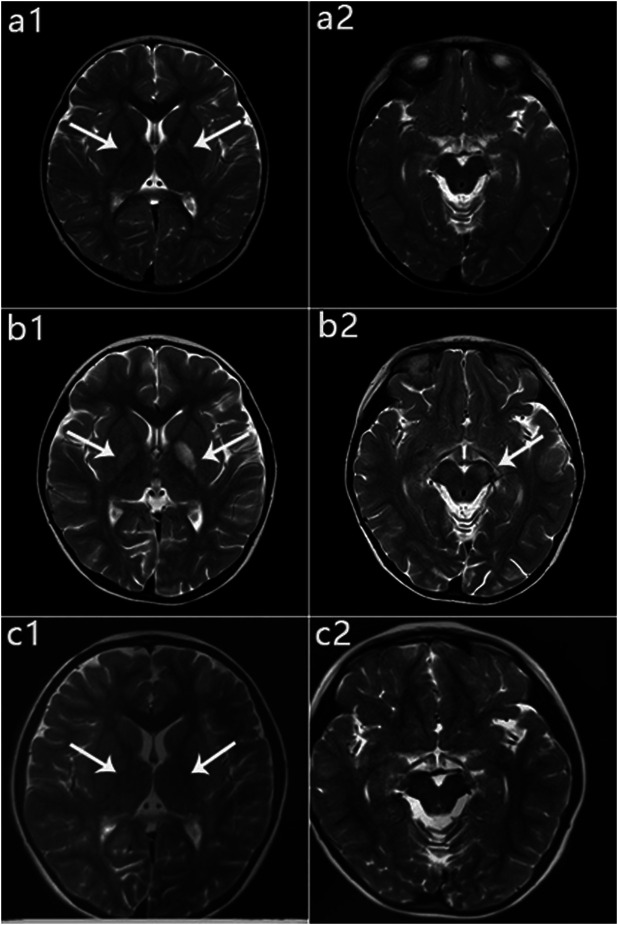
Patient 1: MRI performance in the peak and recovery phases. (a1, a2): Initial MRI obtained at age 2 years; hyperintensity on T2WI in the globus pallidus (a1), normal cerebral peduncle (a2). (b1, b2): MRI performed at 4 years 5 months during the acute stage; hyperintensity on T2WI in the globus pallidus with swelling on the left side (b1) and in the left cerebral peduncle (b2). (c1, c2): MRI performed at 6 years 1 month during the recovery stage; only slightly hyperintensity on T2WI in globus pallidus (c1), and Abnormal signal disappeared in the cerebral peduncle (c2).

The reference for “Charng et al., 2016” was incorrectly written as “Charng, W., Karaca, E., Akdemir, Z. C., Gambin, T., Atik, M. M., Gu, S., et al. (2016). A phenotypically severe, biochemically “silent” case of HIBCH deficiency in a newborn diagnosed by rapid whole exome sequencing and enzymatic testing. Am. J. Med. Genet. 182 (4), 780–784. 10.1002/ajmg.a.61498.” It should be “Charng, W., Karaca, E., Akdemir, Z. C., Gambin, T., Atik, M. M., Gu, S., et al. (2016). Exome sequencing in mostly consanguineous Arab families with neurologic disease provides a high potential molecular diagnosis rate. BMC Med. Genomics 9 (1), 42. 10.1186/s12920-016-0208-3.”

Additionally, there was a mistake in Table 2 as published. The forms of the first row of Table 2 were incorrectly shifted to the left as a whole. The corrected Table 2 appears below.

**TABLE 2:** Metabolite results of extensive investigations of eight patients with *HIBCH* mutations.

	C4-OH (0.00–0.26 μmol/L)			23DH2MB (0.0005–0.0029)		SCPCM (<0.624 μmol/mmol Cr)		Lactic acid	
	Peak phase	Recovery phase	Neonatal period	Peak phase	Recovery phase	Peak phase	Recovery phase	Blood (0.5–2.2 mmol/L)	CSF (1.0–2.78 mmol/L)
Patient 1	0.200	—	—	0.0045↑	—	—	—	1.51	1.46
Patient 2	1.664↑	—	—	0.0715↑	—	8.02↑	—	4.13↑	—
Patient 3	—	0.184	—	—	0.0063↑	—	—	1.60	—
Patient 4	0.579↑	0.183	—	—	0.0123↑	—	2.74↑	0.92	—
Patient 5	0.221	—	—	—	—	—	—	5.23↑	—
Patient 6	0.73↑	0.174	—	0.0032↑	—	2.45↑	—	3.23↑	—
Patient 7	0.485↑	—	—	0.0199↑	—	—	—	1.50	—
Patient 8	1.574↑	1.235↑	0.425↑	—	0.0021	—	—	1.2–2.1	1.50

The authors apologize for this error and state that this does not change the scientific conclusions of the article in any way. The original article has been updated.

