# Supplementary figures and images for: Cinical, Metabolic, and Genetic Analysis and Follow-Up of Eight Patients With HIBCH Mutations Presenting With Leigh/Leigh-Like Syndrome
Source: Front Pharmacol. 2021 Mar 8;12:605803. doi: 10.3389/fphar.2021.605803 (PMC7982470; doi:10.3389/fphar.2021.605803)

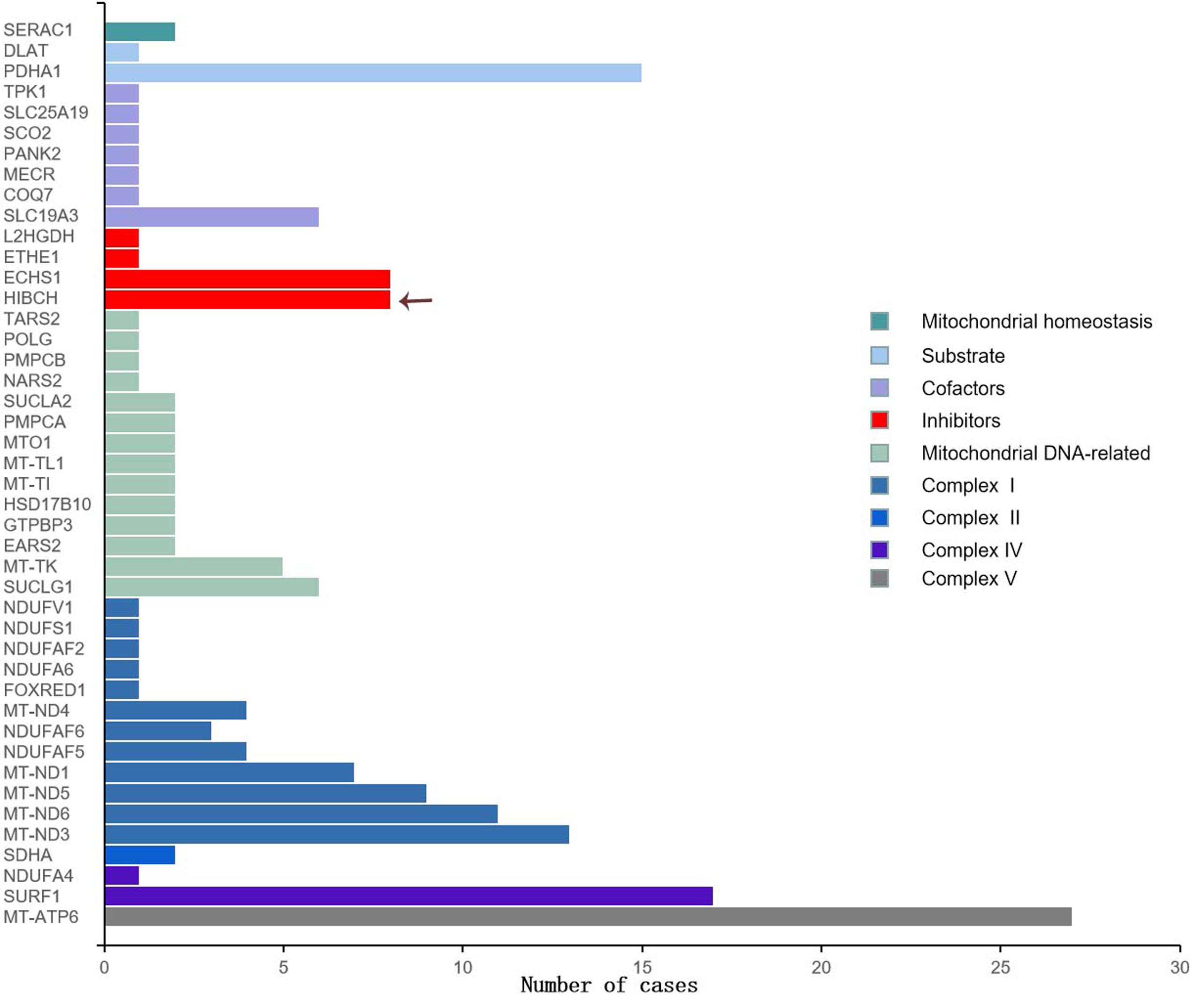

Supplement: Supplementary file 1 [file image1.tif]
