# Supplementary material for: Cinical, Metabolic, and Genetic Analysis and Follow-Up of Eight Patients With HIBCH Mutations Presenting With Leigh/Leigh-Like Syndrome
Source: Front Pharmacol. 2021 Mar 8;12:605803. doi: 10.3389/fphar.2021.605803 (PMC7982470; doi:10.3389/fphar.2021.605803)
Supplement: Supplementary file 2 [file datasheet1.docx]

**Supplementary material:**

**TABLE S1:** ACMG classification of *HIBCH* gene variant sites

**TABLE S2:** Analysis of features between *HIBCH* mutation patients and other 173 patients with Leigh/Leigh-like syndrome

**FIGURE S1:** Genotypes and pathomechanistic features of 181 patients with Leigh/Leigh-like syndrome. The arrow indicates the *HIBCH* mutation.

**TABLE S1 |** ACMG classification of *HIBCH* gene variant sites

| **Patient** | **Nucleotide variation^*^** | **Amino acid variation^*^** | **Reported/Novel** | **ACMG criteria** | **ACMG Classification** |
| --- | --- | --- | --- | --- | --- |
|  |  |  |  |  |  |
| **1** | c.977T>G | p.Leu326Arg | Novel | PM1+PM2+PP3+PP4 | Likely pathogenic |
|  | c.1027C>G | p.His343Asp | Reported | PM2+PM3-Very Strong+PP4 | Pathogenic |
| **2** | c.452C>T | p.Ser151Leu | Reported | PM2+PM3+PP3+PP4 | Likely pathogenic |
|  | c.469C>T | p.Arg157* | Novel | PVS1+PM2+PP4 | Pathogenic |
| **3** | c.750+1G>A | NA | Novel | PVS1+PM2+PP4 | Pathogenic |
|  | c.1036G>T | p.Val346Phe | Novel | PM2+PM3+PP3+PP4 | Likely pathogenic |
| **4** | c.750+1G>A | NA | Novel | PVS1+PM2+PP4 | Pathogenic |
|  | c.1036G>T | p.Val346Phe | Novel | PM2+PM3+PP3+PP4 | Likely pathogenic |
| **5** | c.1027C>G | p.His343Asp | Reported | PM2+PM3-Very Strong+PP4 | Pathogenic |
|  | 868kb deletion Including *HIBCH* | | Novel | PVS1+PM2+PP4 | Pathogenic |
| **6** | c.1027C>G | p.His343Asp | Reported | \| PM21+PM3-Very Strong+PP4 \| \| --- \| \| PM3-Very Stong+PP4 \|   PM2+PM3-Very Stong+PP4 | Pathogenic |
|  | c.439-2A>G | NA | Reported | PVS1+PM2+PP4 | Pathogenic |
| **7** | c.236delC | p.Pro79Leufs*5 | Novel | PVS1+PM2+PP4 | Pathogenic |
|  | c.1027C>G | p.His343Asp | Reported | PM2+PM3-Very Strong+PP4 | Pathogenic |
| **8** | c.810-2A>C | NA | Novel | PVS1+PM2+PP4 | Pathogenic |
|  | c.743A>G | p.His248Arg | Reported | PM2+PM3+PP3+PP4 | Likely pathogenic |

**TABLE S2 |** Analysis of features between *HIBCH* mutation patients and other 173 patients with Leigh/Leigh-like syndrome

|  | ***HIBCH* Group** | **0ther 173 patients Group** |
| --- | --- | --- |
| **predisposing factors** |  |  |
| Infection, n (%) | 4 (50.0) | 53 (30.6) |
| Vaccination, n (%) | 1 (12.5) | 1 (0.6) |
| **Age onset** |  |  |
| 0-1 year, n (%) | 3 (37.5) | 89 (51.4) |
| 1 year- 2 years, n (%) | 8(100.0) | 129(74.6) |
| **Initial symptoms** |  |  |
| Developmental delay, n (%) | 2 (25.0) | 30 (17.3) |
| Developmental regression, n (%) | 2 (25.0) | 50 (28.9) |
| Acute encephalopathy, n (%) | 3 (37.5) | 9 (5.2) |
| Paroxysmal dystonia, n (%) | 1 (12.5) | 17 (9.9) |
| **Main symptoms** |  |  |
| Developmental regression, n (%) | 7 (87.5) | 79 (45.7) |
| Developmental delay, n (%) | 5 (62.5) | 119 (68.8) |
| Seizure, n (%) | 3 (37.5) | 45 (26.0) |
| Ataxia, n (%) | 4 (50.0) | 36 (20.8) |
| Encephalopathy, n (%) | 6 (75.0) | 21 (12.1) |
| Hypotonia, n (%) | 8 (100.0) | 47 (27.2) |
| Dystonia, n (%) | 4 (50.0) | 26 (15.0) |
| Feeding difficulties, n (%) | 5 (62.5) | 33 (19.1) |
| Thyroid dysfunction, n (%) | 2 (25.0) | 5 (2.9) |
| **Metabolites** |  |  |
| Elevated blood lactic acid, n (%) | 3 (37.5) | 123 (71.1) |
| Elevated blood C4-OH, n (%) | 5 (62.5) | 4 (2.3) |
| Elevated urine 23HD2MB, n (%) | 6 (85.7) | 10 (5.8) |
| **Brain imaging involvement** |  |  |
| Basal ganglia, n (%) | 8 (100.0) | 136 (78.6) |
| Brainstem, n (%) | 6 (75.0) | 117 (71.8) |
| Leukodystrophy, n (%) | 3 (37.5) | 30 (17.3) |
| Corpus callosum, n (%) | 1 (12.5) | 3 (1.7) |
| Lactate peak, n (%) | 1 (12.5) | 11 (6.4) |
